# Socio-demographics, neighborhood characteristics, time use, and leisure-time physical activity engagement patterns over the life course

**DOI:** 10.1016/j.ssmph.2022.101244

**Published:** 2022-09-28

**Authors:** Xiaoyue Chen, Astrid Kemperman, Harry Timmermans

**Affiliations:** a Department of the Built Environment, Eindhoven University of Technology, Eindhoven, the Netherlands; b Department of Air Transportation Management, Nanjing University of Aeronautics and Astronautics, Nanjing, China

**Keywords:** Leisure-time physical activity, Life course, Neighborhood characteristics, Multiple discrete-continuous extreme value model, Leisure-time physical activity, LTPA, Recreational walking, cycling, and outdoor play, Recreational-WCP

## Abstract

Physical inactivity remains a major public health challenge today. Understanding the determinants of changes in habitual leisure-time physical activity patterns by type across the life course is important for developing targeted interventions. This study presents a multiple discrete-continuous extreme value model to examine the determinants of habitual participation in and time allocation to multiple leisure-time physical activities over the life course. A comprehensive set of socio-demographics, life transitions, neighborhood characteristics, and time-related factors are considered as determinants of each activity type, including sports, recreational walking, cycling, outdoor playing, and dog walking. Results estimated on retrospective survey data collected in the Netherlands show significant differences in the determinants of the different types of leisure-time physical activity. Social-demographic factors have a strong influence on sports participation, followed by recreational walking, cycling, outdoor playing, and then dog walking. Life transitions have different effects. A change in marital status appears to be the most important life event for sports participation while changing jobs is the most important event for the other two activities. Neighborhood characteristics primarily affect participation in recreational walking, cycling, outdoor playing, and dog walking. As for time-related factors, they mainly impact sports engagement. The findings of this study could help develop effective interventions to promote leisure-time physical activity participation during life transitions and encourage healthy living.

## Highlights

• The determinants of different types of leisure-time physical activity differ significantly.
• Social-demographic factors have a strong impact on sports participation, followed by recreational-WCP, and then dog walking.
• Changes in marital status and work seem to be important events for joining sports and the other two activities, respectively.
• Neighborhood characteristics primarily affect participation in recreational-WCP and dog walking.
• Study-related physical activity time and work-related physical activity time mainly impact sports participation.

## 1 Introduction

Leisure-time physical activity (LTPA) covers different forms of discretionary or recreational bodily movement when one is not working, transporting to a different location, and not doing household chores (2018 Physical Activity Guidelines Advisory Committee, 2018). Sports, recreational walking, cycling, outdoor playing, and dog walking are examples of LTPA categories (2018 Physical Activity Guidelines Advisory Committee, 2018; Merom et al., 2009; Ginis et al., 2012). Its benefits are widely accepted, including reduced risk of chronic diseases (Lahti et al., 2014; Raza et al., 2020), improved social functioning (Shvedko et al., 2018), higher leisure enjoyment (Miller et al., 2016), and greater life satisfaction (Wiese et al., 2018). Therefore, regular participation in LTPA is a crucial component in leading a healthy, active, and enjoyable life.

However, insufficient LTPA remains a major public health challenge. Evidence from 28 European countries revealed that more than one-third of adults were physically inactive (Nikitara et al., 2021), and approximately 1 million deaths in the WHO European Region are attributable to physical inactivity (World Health Organization, 2015). To respond to this challenge, understanding when and how people can be promoted and supported to participate in regular LTPA is thus an important objective.

Previous longitudinal studies have observed that LTPA is complex, multidimensional and fluctuates over the life course (Gropper et al., 2020). Changes in LTPA are predominantly characterized by non-linear decline with age (Jose et al., 2011; Larouche et al., 2012). There seem to be particular periods or transitions during the life course where the decline in LTPA is accelerated or slowed (Gropper et al., 2020; Larouche et al., 2012). Reviews by Engberg et al. (2012) and Allender et al. (2008) concluded that significant changes in LTPA are associated with certain life events, including having children, entering university, returning to study, beginning to work, relocation, and changing marital status, employment status, work conditions, and physical status. Life transitions are often accompanied by shifts between multiple roles, which may disrupt one's daily routine and affect habitual LTPA patterns (Engberg et al., 2012; Gropper et al., 2020). An improved understanding of the dynamic association between life transitions and habitual LTPA patterns over the life course is significant as it may provide a potentially good opportunity for developing sound interventions for those at high-risk.

So far, such studies are still limited and mainly focus on specific time periods or subpopulations, such as early adulthood, women, and older adults. Their results indicated that major life transitions always adversely affect LTPA behavior (Engberg et al., 2012; Wilcox & King, 2004). Specifically, scholars seem to be particularly interested in the period from adolescence to early adulthood, which is associated with a sharp decline in LTPA (Molina-García et al., 2015; Simons et al., 2015). During this life stage, most individuals begin to live independently and face transitions like moving out of home, continuing or discontinuing full-time education, working full-time, developing partner relationships, and becoming a parent (Allender et al., 2008; Winpenny et al., 2020). However, the reported findings appear inconsistent. For example, Simons et al. (2015) suggested that working full-time had no significant associations with LTPA, while Brown and Trost (2003) indicated that it was related to decreased LTPA levels in young women. Besides, a few studies also noticed the influence of a change of social support in this period. Molina-García et al. (2015) found that decreased social support was associated with reductions in LTPA for males.

Research on women has also received much attention, particularly for pregnancy-related and marital status-related changes in LTPA. Consistent evidence was found that LTPA declined and shifted to safer and less vigorous activities throughout pregnancy (Pereira et al., 2007; Poudevigne & O'Connor, 2006). As for marital status transitions of women (i.e., marriage, divorce, widowhood, and remarriage), results show that decreased LTPA was associated with marriage or remarriage, but divorce and widowhood increased LTPA levels (Brown et al., 2009; Lee et al., 2005).

The focus on older adults may stem from the fact that they typically establish new routines with changes in employment, marital status, and physical conditions, which may alter LTPA levels. Most studies agreed that worsening health and widowhood negatively impacted LTPA participation (Droomers et al., 2001; Sawatzky et al., 2007). However, views on the transition to unemployment varied from each other. Slingerland et al. (2007) reported this transition didn't affect LTPA, Lahti et al. (2011) considered it increased moderate-intensity LTPA, while Lee et al. (2020) argued that it specifically decreased male LTPA participation.

Compared with life transitions, other determinants of LTPA are well documented based on numerous cross-sectional studies. Consistent with the ecological models (Sallis et al., 2015), habitual LTPA behavior is the results of a complex interplay of multilevel factors including sociodemographic factors, social support, time use, and built environment factors (Bauman et al., 2012; Giles-Corti & Donovan, 2002). Specifically, participation in LTPA is more common among youngsters (Boutelle et al., 2000; Mitáš et al., 2019), males (Mitáš et al., 2019; Pitsavos et al., 2005), unmarried individuals (Mäkinen et al., 2012; Pitsavos et al., 2005), high-educated people (Mäkinen et al., 2012; Mitáš et al., 2019), individuals in good health (Mäkinen et al., 2012; Orsega-Smith et al., 2007), individuals with supportive social networks (Cho et al., 2021; Lindström et al., 2001; Orsega-Smith et al., 2007), and individuals without overtime work (Kirk & Rhodes, 2011).

Besides, neighborhood attributes were found to promote or discourage the chance of being active (Giles-Corti & Donovan, 2002; Koohsari et al., 2013; Wang et al., 2016). Accessible activity destinations, convenient walking and cycling infrastructure, and safe environments can encourage LTPA participation (Mota et al., 2007; Pereira et al., 2018; Wang et al., 2016). These factors also deserve more attention when exploring LTPA patterns from a life-course perspective. While life transitions may affect LTPA, simultaneous changes in these factors may also alter one's behavior. Therefore, they may be effective in developing interventions to promote LTPA adoption or prevent LTPA decline during life transitions.

Despite these achievements, there is still room for improvement. First, most previous longitudinal studies only examined one or two life transitions or only investigated possible associations within a short lifetime period. A more comprehensive understanding of LTPA participation affected by multiple life transitions based on long-term longitudinal data is needed. Second, previous studies mainly considered total LTPA as the dependent variable and analyzed LTPA at different intensities such as light, moderate, and vigorous. Little research has differentiated the influences of life transition and other determinants within LTPA types. Finally, time spent on physical activity during non-leisure time (e.g., at work or school) may influence LTPA patterns. For instance, Makinen et al. (2009) suggested that individuals in manual occupations were likely to be inactive during leisure time. Studies that explored the impact of time allocated to other physical activity domains on LTPA participation are lacking.

Motivated by the above discussion, this study aimed to explore the effect of socio-demographics, life transitions, neighborhood characteristics, and time-related factors on habitual LTPA patterns by type over the life course. Specifically, this study focused on three central questions:1) Are there differences in the choice of LTPA types among groups with different characteristics? 2) Do determinants, including socio-demographics, life transitions, neighborhood characteristics, and time-related factors, affect changes in time allocation decisions for habitual LTPA patterns? 3) How does each determinant work differently for multiple types of leisure-time physical activity participation? Based on retrospective longitudinal data collected online in the Netherlands, this study adopted a multiple discrete-continuous extreme value (MDCEV) model to examine the determinants of the choice of and allocation of time to LTPA types including sports, recreational walking, cycling, and outdoor play (Recreational-WCP), and dog walking. The MDCEV model was developed by Bhat (Bhat, 2005, 2008) and can jointly model people's activity choices (the discrete choice alternatives, the LTPA types) as well as the time invested in each of the chosen activities (the continuous-time allocation decision). The results may expand our understanding of the determinants of LTPA pattern changes from a life-course perspective and help develop effective interventions to promote LTPA participation.

## 2 Method

### 2.1 MDCEV model

MDCEV model originates from a utility-theoretic and satiation view of time-use and is increasingly applied to activity-based behavior research (e.g., Paleti et al., 2011; Pinjari et al., 2009; Sener & Bhat, 2012). The model is ideally applicable to this study because it can address the issue of participating in LTPA types, jointly with modelling the weekly participation time of each activity. In this study, LTPA was categorized into three types: (1) Sports, (2) Recreational-WCP, and (3) Dog walking. Without loss of generality, this study also defines another “activities outside LTPA” category with a time allocation computed as the difference between the total hours in a week and the time allocated to LTPA, which constitutes the “outside good” in the MDCEV model system. The “outside good” means a good that is always consumed by all consumers. Here, it refers to activities that people must invest time in, such as working, studying, and sleeping. This section outlines the MDCEV model structure, more details can be found in Bhat's research (Bhat, 2005, 2008).

Let *k* represent the index for the four alternatives. Let *t*_*k*_ (*k* = 1) be the non-zero amount of time invested in the activities outside LTPA and let *t*_*k*_ (*k* = 2,3, or 4) be the time invested in LTPA alternative *k*. The overall random utility function takes the following form:

(1) $$U\left(t\right)=\frac{1}{\alpha_{1}}\exp\left(\varepsilon_{1}\right)t_{1}^{\alpha_{1}}+\sum_{k=2}^{K}\frac{\gamma_{k}}{\alpha_{k}}\left[\exp\left(\beta^{\prime }z_{k}+\varepsilon_{k}\right)\right]\left\{{\left(\frac{t_{k}}{\gamma_{k}}+1\right)}^{\alpha_{k}}-1\right\}$$

where, **z**_k_ is a vector of exogenous determinants (including a constant) specific to alternative *k*. The determinants of this study were extracted from the literature and initially screened by correlation tests: highly correlated determinants were removed. ε_k_ captures the unobserved characteristics that impact the baseline utility for alternative *k*. As the baseline preference, the term exp(**β′z**_**k**_+ε_k_) represents the random marginal utility for alternative *k* at the point of zero-time investment for the alternative. It controls the discrete choice participation decision in alternative *k*. ***β′*** is a vector of parameters to be estimated, which works as preference weights. α_k_ (α_k_ ≤ 1) is a satiation parameter which reduces the marginal utility with increasing time investment of alternative *k*. γ_k_ is a translation parameter that allows corner solutions (i.e., no time investment in activity *k*). It also serves as satiation parameters, but is inversely associated with satiation for alternative *k*. There is no γ_1_ term because all individuals invest some time in activities outside LTPA. Note that the analyst will generally be unable to estimate both α_k_ and γ_k_ for the alternative *k* (*k* ≥ 2). Under the restrictions, this study has estimated the following five utility forms suggested by Bhat and selected the one that fits the data best as the final model through Akaike's information criteria (AIC) and Bayesian information criteria (BIC). Eq. (4) is estimable because α is a constant and obtains a “pinning effect” from the satiation parameter for the activities outside LTPA alternative.

(2) $$U\left(t\right)=\frac{1}{\alpha_{1}}\exp\left(\varepsilon_{1}\right)t_{1}^{\alpha_{1}}+\sum_{k=2}^{K}\frac{1}{\alpha_{k}}\left[\exp\left(\beta^{\prime }z_{k}+\varepsilon_{k}\right)\right]\left\{{\left(t_{k}+1\right)}^{\alpha_{k}}-1\right\}$$

(3) $$U\left(t\right)=\frac{1}{\alpha_{1}}\exp\left(\varepsilon_{1}\right)t_{1}^{\alpha_{1}}+\sum_{k=2}^{K}\gamma_{k}\left[\exp\left(\beta^{\prime }z_{k}+\varepsilon_{k}\right)\right]\ln\left(\frac{t_{k}}{\gamma_{k}}+1\right)$$

(4) $$U\left(t\right)=\frac{1}{\alpha }\exp\left(\varepsilon_{1}\right)t_{1}^{\alpha }+\sum_{k=2}^{K}\frac{\gamma_{k}}{\alpha }\left[\exp\left(\beta^{\prime }z_{k}+\varepsilon_{k}\right)\right]\left\{{\left(\frac{t_{k}}{\gamma_{k}}+1\right)}^{\alpha }-1\right\}$$

(5) $$U\left(t\right)=\exp\left(\varepsilon_{1}\right)\ln\left(t_{1}\right)+\sum_{k=2}^{K}\left[\exp\left(\beta^{\prime }z_{k}+\varepsilon_{k}\right)\right]\ln\left(t_{k}+1\right)$$

(6) $$U\left(t\right)=\exp\left(\varepsilon_{1}\right)\ln\left(t_{1}\right)+\sum_{k=2}^{K}\gamma_{k}\left[\exp\left(\beta^{\prime }z_{k}+\varepsilon_{k}\right)\right]\ln\left(\frac{t_{k}}{\gamma_{k}}+1\right)$$

The individual's maximized random utility *U*(*t*) is constrained by the activity time budget that $\sum_{k=1}^{K}t_{k}=T$, where *T* is the total time available for the individual to participate in the four activities. *T* for this study equals 168 h, the total number of hours in a week. The optimal time investments $t_{k}^{*}$ (*k* = 1, 2, …, *K*) can be found by forming the Lagrangian function and applying the Kuhn-Tucker (KT) conditions. Assuming the error terms ε_k_ (*k* = 1, 2, …, *K*) are independent and identically distributed across alternatives with a type-1 extreme value distribution, the probability expression for the time investment in the first *M* of the *K* alternatives is:

(7) $$P\left(t_{1}^{*},t_{2}^{*},\ldots ,t_{M}^{*},0,0,\ldots ,0\right)=\left\lfloor \prod_{i=1}^{M}c_{i}\right\rfloor \left\lfloor \sum_{i=1}^{M}\frac{1}{c_{i}}\right\rfloor \left\lceil \frac{\prod_{i=1}^{M}e^{V_{i}}}{\left(\sum_{k=1}^{K}e^{V_{k}}\right)}\right\rceil \left(M-1\right)!$$

Where $c_{1}=\left(\frac{1-\alpha_{1}}{t_{i}^{*}}\right)\;and$ $c_{i}=\left(\frac{1-\alpha_{i}}{t_{i}^{*}+\gamma_{i}}\right)$ for i = 2, …, M.

The expressions for *V* in Eq. (7) are as follows for the estimated five utility forms:

$$For\;Eq.\;\left(2\right):\;V_{1}=\left(\alpha_{1}-1\right)\ln\left(t_{k}^{*}\right);\;V_{k}=\beta^{\prime }z_{k}-\left(\alpha_{k}-1\right)\ln\left(t_{k}^{*}+1\right)\left(k\geq 2\right)$$

$$For\;Eq.\;\left(3\right):\;V_{1}=\left(\alpha_{1}-1\right)\ln\left(t_{i}^{*}\right);\;V_{k}=\beta^{\prime }z_{k}-\ln\left(\frac{t_{k}^{*}}{\gamma_{k}}+1\right)\left(k\geq 2\right)$$

$$For\;Eq.\;\left(4\right):\;V_{1}=\left(\alpha_{1}-1\right)\ln\left(t_{1}^{*}\right);\;V_{k}=\beta^{\prime }z_{k}+\left(\alpha -1\right)\ln\left(\frac{t_{k}^{*}}{\gamma_{k}}+1\right)\left(k\geq 2\right)$$

$$For\;Eq.\;\left(5\right):\;V_{1}=-\ln\left(t_{1}^{*}\right);\;V_{k}=\beta^{\prime }z_{k}-\ln\left(t_{k}^{*}+1\right)\left(k\geq 2\right)$$

$$For\;Eq.\;\left(6\right):\;V_{1}=-\ln\left(t_{1}^{*}\right);\;V_{k}=\beta^{\prime }z_{k}-\ln\left(\frac{t_{k}^{*}}{\gamma_{k}}+1\right)\left(k\geq 2\right)$$

The parameters can be estimated through a maximum likelihood approach. This study used R software to estimate the model.

### 2.2 Data source and sample

#### 2.2.1 Survey design

A web-based retrospective survey developed by the authors was used to collect data for this study. Retrospective surveys are backward-directional design and gather information from records or memories. Despite a risk of memory bias, they are reliable for life-course research because evidence has shown that people can always accurately recall the occurrence and timing of important events they experienced (Moschis, 2019). With the advantages of being comparatively inexpensive, less time-consuming, and covering longer time spans, retrospective surveys have become a useful tool for collecting life-course and behavior data (Moschis, 2019). As this study tends to understand habitual LTPA patterns under the influence of major life transitions over the long-term lifespan, the retrospective survey is appropriate.

The survey mainly consists of two sections: life course and physical activity. Questions for each life event in the life-course section first asked respondents to indicate whether they have experienced the event (or their current status) and then, if they have, give a detailed chronological description from birth to today. The investigated life events included changes in marital status, household composition, home location, education, employment, diagnosis of chronic diseases, living arrangements with physically active people, and car ownership. Note that information about neighborhood characteristics was co-recorded with each home location. Respondents were asked to assess neighborhood characteristics on a Likert five-point scale with options including few, below average, the average amount for a typical neighborhood, above average, and a lot. Also, weekly hours spent on active commuting, physical education, and physical activity at work were co-recorded with each educational and working experience. Table 1 includes detailed information on each life-course question.

**Table 1:** Information on data collection of life-course trajectory and habitual physical activity behaviors.

Question categories	Information collected in the survey
**Major life events**	
Marital status	- Current marital status including single, living together, registered partnership, married, divorced, widowed, and others - History of marital status change including change time and new status after the change
Baby birth	- Number of children - Information of each child including the birth year and gender
Relocation	- The earliest home address (since birth year) - History of relocation including moving time and new address - Information of neighborhood characteristics for each home address, and the characteristics include greenspace for walking, cycling facilities, physical activity facilities, and safety for doing physical activity
Education	- Education background including start time, end time, school location, education level, weekly physical education hours - For each school, collect commuting information between home and school, including start time, end time, mode of transportation, frequency, and daily commute time
Employment	- History of employment including start time, end time, weekly working hours, company location, and weekly physical activity time at work - For each job, collect commuting information between home and workplace, including start time, end time, mode of transportation, frequency, and daily commute time
Chronic diseases diagnosis (self)	The respondent's own chronic disease history, including the start time and end time
Chronic diseases diagnosis (family)	Chronic diseases history of the respondent's parents/partner/children, including the start time and end time
Living arrangements with physically active people	History of living with someone who regularly did physical activities, and the information includes start time, end time, and relationship
Car ownership	- Number of cars - Ownership duration of each car (start time and end time)
**Habitual physical activity behaviors**	
Sports	For each sport played on a regular basis, collect the following information: start time, end time, sport type, weekly activity time (h/w), and motivations to start.
Shopping for daily necessities	For each period of shopping for daily necessities on a regular basis, collect the following information: start time, end time, shopping frequency, mode of transportation to stores, round-trip travel time (mins/round-trip), and weekly shopping time (h/w).
Shopping for non-daily products	For each period of shopping for non-daily products on a regular basis, collect the following information: start time, end time, shopping frequency, mode of transportation to stores, round-trip travel time (mins/round-trip), and shopping time per month (h/m).
Recreational walking, cycling and outside playing	For each period of walking, cycling, and outside playing for fun on a regular basis, collect the following information: start time, end time, weekly activity time (h/w), and motivations to start.
Dog walking	For each period of dog walking on a regular basis, collect the following information: start time, end time, frequency, and weekly activity time (h/w).
Housework	For each period of doing housework on a regular basis, collect the following information: start time, end time, frequency, and weekly activity time (h/w).
Gardening	For each period of regular gardening in the spring or summer, collect the following information: start year, end year, frequency, activity time in spring, and activity time in summer.

To measure physical activity, respondents were first asked to indicate whether they have regularly participated (participated in at least once a week and lasted more than half a year) in one or more of the following activities: sports, shopping for daily necessities, shopping for non-daily products, recreational-WCP, dog walking, housework, and gardening. According to the responses, respondents were asked for more details on each activity they regularly participated in, including start time, end time, frequency, and weekly activity hours. Table 1 presents the details of each physical activity question. Since this study focused on LTPA patterns, the further analysis only extracted information on sports, recreational-WCP, and dog walking.

The survey was created and run using Limesurvey, a professional online survey tool. It allows users to design questions and check procedures through programming freely. To minimize the possible bias from memory, the created online survey system contained many error-checking functions to guarantee consistency in responses. Firstly, the system checked the consistency of the provided current and historical information. Respondents were triggered to check when the events they reported didn't logically lead to their current situation. Secondly, the system checked the temporal logic of life events and behaviors. For example, the system reminded respondents to check when the end time was before the start time. Finally, the system checked the logic of transitions. For instance, if at some stage the respondent reported being single, the next stage should not be single, divorced, or widowed.

#### 2.2.2 Data collection

Data for this study were collected in the Netherlands through a retrospective online survey between September and October 2020. The data collection process was done in collaboration with Panelclix, a national survey company which maintains a representative panel of the Dutch population. Their panelists can be approached specifically (representative and stratified) based on the characteristics they have provided to the survey company. The survey company sends invitations to all the qualifying panelists and closes the survey when the target number is reached. The qualifying panelists of this survey were representative for the Dutch population over the age of 18 years old. A total of 627 panelists completed the survey.

#### 2.2.3 Generate activity episodes for analysis

As this study sought to simultaneously evaluate changes in LTPA patterns (participation in activity types and weekly activity duration) and associated life transitions, which are dynamic and may occur multiple times over the life course, LTPA data were integrated with the life-course data. Numerous activity episodes were generated for each respondent for analysis. For each life event, the weekly time investment of each activity when the life event occurred were recorded. Co-occurrence is assumed to indicate that the life transition directly or indirectly triggered the LTPA pattern. Note that the impact of a life transition on LTPA patterns may be delayed, so LTPA data of the new pattern was recorded if LTPA patterns changed within a certain period after the life transition. For baby birth, the period was assumed to be one year, while for others, it was assumed to be six months. Besides, data reflecting changes in LTPA patterns but without the occurrence of a life event were also recorded. In addition to life transitions, data on other determinants were also appended when life events or LTPA patterns changed to compile a comprehensive dataset for modeling. Finally, 8398 activity episodes were generated for analysis.

### 2.3 Exogenous determinants

The exogenous variables considered in the model specification included four types: (1) socio-demographics (age, gender, have a partner, education level, car ownership, have chronic diseases, and live with physically active people), (2) life transitions (marital status change, baby birth, relocation, change schools, stop full-time education, start working, change jobs, and own the first car), (3) neighborhood characteristics (greenspace for walking, cycling facilities, supportive facilities for physical activities, and safety for physical activities), and (4) time-related factors (study-related physical activity time, and work-related physical activity time). Table 2 presents the distribution of all the explanatory variables considered in the model specification. Effect coding was used to represent all the categorical variables. This coding method represents group membership with dummy variables that take on the values 1, 0, and −1. Specifically, membership in a particular group is coded 1, non-membership in the group is coded 0, whereas the reference group is coded −1.

**Table 2:** Descriptive statistics of explanatory variables (N = 8398).

Variable	Categories	Frequency (%)	Participation rate by activity type (%)		
			Sports	Recreational walking, cycling, and outdoor play	Dog walking
Age	≤7	854 (10.2%)	15.7%	8.3%	1.4%
	7–23	4340 (51.7%)	32.3%	20.9%	4.6%
	>23	3204 (38.1%)	28.3%	46.7%	8.8%
Gender	Female	4179 (49.8%)	27.4%	28.1%	5.7%
	Male	4219 (50.2%)	30.8%	30.7%	6.0%
Have a partner or not	Yes	2468 (29.4%)	70.9%	56.5%	11.5%
	No	5930 (70.6%)	11.7%	18.2%	3.5%
Education level	Primary or lower	2431 (28.9%)	22.9%	16.7%	5.1%
	Secondary	3679 (43.8%)	26.7%	30.9%	6.8%
	Higher	2299 (27.2%)	39.4%	40.6%	5.0%
Car ownership	Have a car	2843 (33.9%)	32.7%	41.4%	9.4%
	Have no cars	5555 (66.1%)	27.3%	23.3%	4.1%
Have chronic diseases or not (Self)	Yes	763 (9.1%)	23.1%	41.0%	10.6%
	No	7635 (90.9%)	29.7%	28.3%	5.4%
Have chronic diseases or not (Family)	Yes	916 (10.9%)	32.6%	40.2%	6.3%
	No	7482 (89.1%)	28.7%	28.1%	5.7%
Live with physically active people or not	Yes	1230 (14.6%)	48.9%	41.5%	7.6%
	No	7168 (85.4%)	25.7%	27.4%	5.6%
Marital Status change	Yes	586 (7.0%)	24.7%	34.8%	5.6%
	No	7812 (93.0%)	29.4%	29.0%	5.9%
Baby birth	Yes	574 (6.8%)	21.6%	40.8%	6.8%
	No	7824 (93.2%)	29.6%	28.6%	5.8%
Relocation	Yes	1084 (12.9%)	25.0%	29.7%	4.6%
	No	7314 (87.1%)	29.7%	29.4%	6.0%
Change schools	Yes	2290 (27.3%)	26.3%	14.3%	2.4%
	No	6108 (72.7%)	30.1%	35.1%	7.2%
Stop full-time edcuation	Yes	635 (7.6%)	25.7%	25.8%	4.3%
	No	7763 (92.4%)	29.4%	29.7%	6.0%
Start working	Yes	646 (7.7%)	28.9%	30.3%	4.6%
	No	7752 (92.3%)	29.1%	29.4%	5.9%
Change jobs	Yes	725 (8.6%)	26.6%	42.6%	6.9%
	No	7673 (91.4%)	29.3%	28.2%	5.7%
Own the first car	Yes	519 (6.2%)	26.8%	28.7%	5.8%
	No	7879 (93.8%)	29.2%	29.5%	5.9%
Greenspace for walking	Below average	1633 (19.4%)	27.7%	28.2%	4.8%
	Average	2930 (34.9%)	29.5%	27.6%	5.4%
	Above average	3835 (45.7%)	29.3%	31.4%	6.6%
Cycling facilities	Below average	1351 (16.1%)	22.2%	27.7%	8.6%
	Average or above average	6148 (73.2%)	30.8%	29.1%	4.7%
	A lot	899 (10.7%)	27.8%	34.1%	9.2%
Supportive facilties for physical activities	Below average	1634 (19.5%)	26.6%	26.6%	6.7%
	Average or above average	6230 (74.5%)	29.8%	30.6%	5.1%
	A lot	534 (6.4%)	28.1%	24.5%	12.2%
Safety for physical activities	Average or Below average	2996 (35.7%)	27.3%	26.8%	7.4%
	Above average	4212 (50.2%)	29.7%	32.0%	4.8%
	Very safe	1190 (14.2%)	31.3%	27.0%	5.6%
Study-related physical activity time	0h/w	5800 (69.1%)	28.2%	34.8%	6.5%
	0–2.5h/w	580 (6.9%)	28.4%	17.2%	5.7%
	2.5–5h/w	929 (11.1%)	30.8%	16.9%	4.5%
	>5h/w	1089 (13.0%)	32.6%	18.2%	3.4%
Work-related physical activity time	0h/w	5751 (68.5%)	27.9%	24.5%	5.1%
	0–2.5h/w	779 (9.3%)	36.1%	44.2%	7.4%
	2.5–5h/w	544 (6.5%)	34.2%	44.9%	6.8%
	>5h/w	1324 (15.8%)	28.1%	36.1%	7.6%

**Fig. 1:**
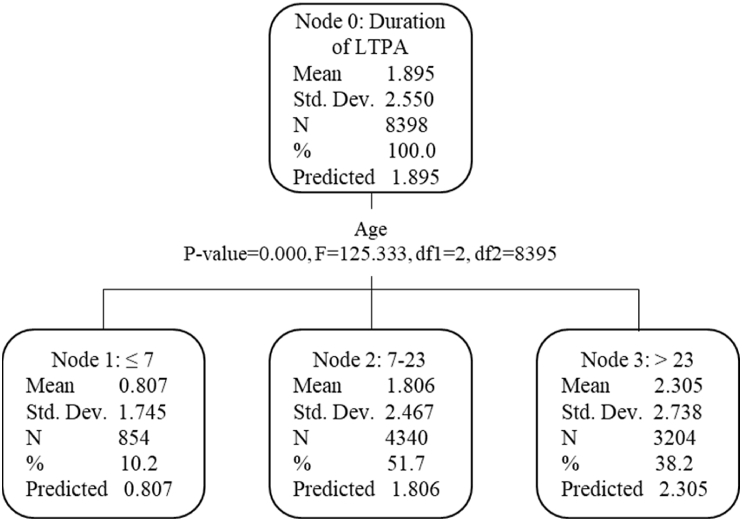
Results of decision tree (Age).

Some annotations on these variables are necessary here. Firstly, Age categories were defined according to the results of the decision tree (Fig. 1). Secondly, judgments of having a partner were derived from respondents’ responses to marital status at each life stage, categorizing single, divorced, and widowed as having no partner, and living together, registered partnership, and married as having a partner. This classification was based on the different living arrangement forms in the Netherlands. Thirdly, this study believed that chronic diseases of respondents themselves or having family members with chronic diseases might have different effects on LTPA patterns, so they were included in the analysis separately. Fourthly, neighborhood characteristics were assessed in the survey on a Likert five-point scale but reclassified into three levels based on similarity in response distributions (see Table 2). Cycling facilities referred to the designated spaces and facilities for cyclists like cycle paths and bicycle parking. Supportive facilities for physical activities referred to the designated spaces and facilities like courts, fitness equipment, and playgrounds. Safety for physical activities involves personal perceptions of neighborhood traffic safety, crime risks, and sports injuries. Finally, study and work-related physical activity time, measured in hours per week, was the sum of physical activity time at school (physical education) or at work and active commute time (walking and cycling).

## 3 Results

### 3.1 Descriptive statistics of respondents

Demographics of respondents showed that 85% of them regularly participated in at least one LTPA. The percentages of respondents aged 18–29, 30–39, 40–54, and over 55 were 25.8%, 26.3%, 26.5%, and 21.4%, respectively. Males (52.3%) and females (47.7%) were more or less equally represented. About 18.3% of the respondents had a primary or lower education level, 39.2% had a secondary education level, and 42.42% had a higher education level. Based on the Dutch education system, secondary education comprised pre-vocational secondary education (VMBO), senior general secondary education (HAVO), pre-university education (VWO), secondary vocational education (MBO), and other equivalent education. Higher education comprised higher professional education (HBO-bachelor/master), university education (WO-bachelor/master/doctor), and other equivalent or higher education.

### 3.2 Descriptive statistics of activity episodes

Table 3 provides a summary of LTPA participation by type. About 50.7% participated in at least one LTPA type. The highest participation rate was observed in recreational-WCP, and the lowest was in dog walking. The mean duration for sports was the longest, about 3.74h/w.

**Table 3:** Descriptive statistics of LTPA participation (N = 8398).

Activity	Total number (%) of participation	Mean duration of participation among those who participated in the activity (h/w)	Number of participation … (% of total participants)	
			Only in this activity type	Also in other activity types
Leisure-time physical activities:	4259 (50.7%)	1.89		
• Sports	2443 (29.1%)	3.74	1538 (63%)	905 (37%)
• Recreational walking, cycling, and outdoor play	2473 (29.4%)	2.31	1473 (59.6%)	1000 (40.4%)
• Dog walking	491 (5.8%)	2.17	188 (38.3%)	303 (61.7%)
Activities outside LTPA	8398 (100%)	166.12	4139 (49.3%)	4259 (50.7%)

As shown in Table 2, the age distribution indicated that the under-23 age group had the highest sports participation rate, while the over-23 age group had the highest recreational-WCP participation rate. Males had slightly higher participation rates than females across all LTPA types. Similarly, those who had a partner, owned cars, lived with physically active people, lived in a neighborhood with above-average green spaces, and those whose family members were unhealthy showed higher levels of engagement across all LTPA types. Conversely, people with experienced school changes or cessation of full-time education had lower participation rates across all LTPA types than those without these changes. For the categories of other variables, their performance in terms of participation rates varied by activity type, implying that differences may exist in their effects on different activity types.

### 3.3 Results of the MDCEV model

Results of the model comparison are listed in Table 4. With the lowest AIC and BIC values, the last utility form Eq. (6) provides the best fit and is therefore selected as the final model. Table 5 shows the MDCEV model results, and the following sections offer a detailed discussion of the findings. The outside good, activities outside LTPA, serves as the base alternative in the model estimation. The estimates presented in the table refer to the *β* vector in Eq. (6).

**Table 4:** Comparison of models based on different utility forms.

Utility forms	Log likelihood	df	AIC	BIC
Eq. (2)	−23894.65	103	47995.3	48719.98
Eq. (3)	−23547.8	103	47301.59	48026.27
Eq. (4)	−23547.8	103	47301.59	48026.27
Eq. (5)	−23919.9	99	48037.8	48734.34
Eq. (6)	**−23547.8**	**102**	**47299.6**	**48017.25**
Model with only baseline preference constants and the satiation parameters	−24619.19	6	49250.39	49292.60

**Table 5:** The results of MDCEV model estimation.

Variable	Categories	Sports		Recreational walking, cycling, and outdoor play		Dog walking		Activities outside LTPA	
		Coeff.	P-value	Coeff.	P-value	Coeff.	P-value	Coeff.	P-value
**Socio-demographics**									
Age	≤7	−4.6388e-01	–	−6.4398e-01	–	−1.039e+00	–	–	–
	7–23	4.492e-01	0.000***	1.818e-02	0.744	3.600e-01	0.004**	–	–
	>23	1.468e-02	0.820	6.258e-01	0.000***	6.794e-01	0.000***	–	–
Gender	Female	−1.412e-01	–	−2.630e-03	–	1.100e-02	–	–	–
	Male	1.412e-01	0.000***	2.630e-03	0.923	−1.100e-02	0.849	–	–
Have a partner	Yes	−1.116e-01	0.003**	6.282e-02	0.062	7.083e-02	0.273	–	–
	No	1.116e-01	–	−6.282e-02	–	−7.083e-02	–	–	–
Education level	Primary or lower	−2.1e-01	–	−3.1e-01	–	2.023e-01	–	–	–
	Secondary	−1.599e-01	0.000***	1.151e-01	0.002**	1.719e-01	0.016*	–	–
	Higher	3.699e-01	0.000***	1.949e-01	0.000***	−3.742e-01	0.000***	–	–
Car ownership	Yes	1.566e-01	0.000***	4.993e-02	0.125	3.466e-01	0.000***	–	–
	No	−1.566e-01	–	−4.993e-02	–	−3.479e-01	–	–	–
Have chronic diseases (Self)	Yes	−2.037e-01	0.000***	9.090e-02	0.032*	1.605e-01	0.032*	–	–
	No	2.037e-01	–	−9.090e-02	–	−1.605e-01	–	–	–
Have chronic diseases (Family)	Yes	1.301e-01	0.003**	1.692e-01	0.000***	−1.341e-02	0.877	–	–
	No	−1.301e-01	–	−1.692e-01	–	1.341e-02	–	–	–
Live with physically active people	Yes	4.257e-01	0.000***	1.270e-01	0.000***	6.458e-02	0.341	–	–
	No	−4.257e-01	–	−1.270e-01	–	−6.458e-02	–	–	–
**Life transitions**									
Marital Status change	Yes	−2.240e-01	0.000***	−1.578e-01	0.002**	−4.023e-01	0.000***	–	–
	No	2.240e-01	–	1.578e-01	–	4.023e-01	–	–	–
Baby birth	Yes	−3.823e-01	0.000***	−1.749e-01	0.001***	−4.054e-01	0.000***	–	–
	No	3.823e-01	–	1.749e-01	–	4.054e-01	–	–	–
Relocation	Yes	−2.707e-01	0.000***	−1.937e-01	0.000***	−4.136e-01	0.000***	–	–
	No	2.707e-01	–	1.937e-01	–	4.136e-01	–	–	–
Change schools	Yes	−2.523e-01	0.000***	−3.563e-01	0.000***	−5.692e-01	0.000***	–	–
	No	2.523e-01	–	3.563e-01	–	5.692e-01	–	–	–
Stop full-time edcuation	Yes	−2.993e-01	0.000***	−2.343e-01	0.000***	−4.049e-01	0.000***	–	–
	No	2.993e-01	–	2.343e-01	–	4.049e-01	–	–	–
Start working	Yes	−2.306e-01	0.000***	−1.876e-01	0.000***	−4.365e-01	0.000***	–	–
	No	2.306e-01	–	1.876e-01	–	4.365e-01	–	–	–
Change jobs	Yes	−2.862e-01	0.000***	−1.134e-01	0.019*	−3.042e-01	0.001***	–	–
	No	2.862e-01	–	1.134e-01	–	3.042e-01	–	–	–
Have the first car	Yes	−3.476e-01	0.000***	−2.905e-01	0.000***	−5.826e-01	0.000***	–	–
	No	3.476e-01	–	2.905e-01	–	5.826e-01	–	–	–
**Neighborhood characteristics**									
Greenspace for walking	Below average	4.8879e-02	–	−2.358e-02	–	−4.133e-01	–	–	–
	Average	−5.279e-03	0.892	−7.034e-02	0.086	1.627e-01	0.053	–	–
	Above average	−4.360e-02	0.277	9.392e-02	0.022*	2.506e-01	0.002**	–	–
Cycling facilities	Below average	−2.2602e01	–	−4.71e-02	–	3.2216e-01	–	–	–
	Average or above average	1.717e-01	0.000***	−1.089e-01	0.017*	−3.675e-01	0.000***	–	–
	A lot	5.432e-02	0.429	1.560e-01	0.025*	4.534e-02	0.709	–	–
Supportive facilities for physical activities	Below average	3.914e-02	–	8.990e-02	–	−2.564e-01	–	–	–
	Average or above average	−1.226e-04	0.998	1.918e-01	0.000***	−2.938e-01	0.001***	–	–
	A lot	−3.902e-02	0.639	−2.817e-01	0.001***	5.502e-01	0.000***	–	–
Safety for physical activities	Average or Below average	−3.6671e-02	–	−6.480e-02	–	4.323e-01	–	–	–
	Above average	2.561e-03	0.947	1.139e-01	0.004*	−2.200e-01	0.010*	–	–
	Very safe	3.411e-02	0.532	−4.910e-02	0.388	−2.123e-01	0.076	–	–
**Time-related factors**									
Study-related physical activity time	0h/w	−1.51e-01	–	2.1001e-01	–	−1.6765e-01	–	–	–
	0–2.5h/w	−2.195e-01	0.008**	−2.787e-01	0.004**	3.688e-01	0.027*	–	–
	2.5–5h/w	2.036e-01	0.003**	4.504e-02	0.584	9.845e-02	0.505	–	–
	>5h/w	1.669e-01	0.008**	2.365e-02	0.752	−2.996e-01	0.054	–	–
Work-related physical activity time	0h/w	−8.279e-02	–	1.059e-01	–	−5.503e-02	–	–	–
	0–2.5h/w	1.757e-01	0.013*	3.370e-03	0.959	1.387e-02	0.910	–	–
	2.5–5h/w	7.279e-02	0.361	1.033e-01	0.164	−2.106e-02	0.886	–	–
	>5h/w	−1.657e-01	0.006**	−8.026e-04	0.989	6.222e-02	0.557	–	–
Baseline preference constants		−7.836e+00	0.000***	−7.524e+00	0.000***	−1.060e+01	0.000***	–	–
Translation parameters-$\gamma_{k}$		2.324e+00	0.000***	1.161e+00	0.000***	1.298e+00	0.000***	–	–

Significance codes: p < 0.001***; p < 0.01**; p < 0.05.

#### 3.3.1 Socio-demographics

Age, education level and having chronic diseases (self) have significant effects on all LTPAs, but the effect varies by type. Specifically, results show an increased likelihood of dog walking with age and reveal that people at 7–23 years are more likely to engage in sports, while those over 23 years are more likely to engage in recreational-WCP. It is consistent with the Sport and Exercise Behavior Report (Duijvestijn et al., 2021), indicating most Dutch people discontinued weekly sports at 17–18 years old.

Additionally, people with higher education are most likely to do sports, followed by recreational-WCP, but are less likely to walk dogs. Those with a secondary education are more likely to participate in recreational-WCP and dog walking but are less likely to play sports. And lower educated people are most likely to walk dogs but less likely to engage in the other LTPA types. This result may be related to occupation. Previous studies have proven that occupational categories are directly associated with LTPA, with white-collar showing the higher LTPA levels compared to blue-collar workers (Kirk & Rhodes, 2011).

The estimated parameters of having chronic diseases (self) display that people with the illness are more likely to be active in recreational-WCP, and dog walking but less likely to play sports. Regular participation in LTPA could help people with chronic illnesses recover (Lahti et al., 2014; Raza et al., 2020). Due to physical constraints, high-intensity sports may not be good options for the patients, and moderate- or light-intensity LTPAs like recreational walking, cycling, and outdoor play are more appropriate.

Regarding other social-demographic variables, gender and having a partner only significantly affect sports participation, i.e., males and those without a partner are more likely to play sports. Owning a car is positively associated with participating in sports and dog walking, which may be related to good economic conditions. As expected, people who live with physically active people or whose family members have chronic diseases are more likely to engage in sports and recreational-WCP. It reflects that social support from peers and family, increased health concerns, and improved awareness of LTPA benefits may play an important role in LTPA participation.

#### 3.3.2 Life transitions

All life transition variables show significant results for all LTPA types, albeit the estimated parameters are negative. It suggests that people are less likely to participate in LTPAs when an event occurs. It is consistent with previous studies discussed in the Introduction. Nevertheless, the estimates still illustrate the differential impact of various life transitions on LTPA patterns. The impact of school changes is special. Its estimates for LTPA types, in descending order, are 1) sports, 2) recreational-WCP, and 3) dog walking. But for other life transition variables, the estimates, in descending order, are 1) recreational-WCP, 2) sports, and 3) dog walking. That is, people are more likely to play sports when transferring schools but are more likely to be active in recreational-WCP when other life events occur. It fits the estimates of age. School transfers mostly occur in the 7–23 age group and are more likely to participate in sports than other LTPA types. While other life transitions primarily occur in the over-23 age group, who are more likely to engage in recreational-WCP.

Moreover, attention should be paid to the association between life transitions and each LTPA type. Estimates provide that changing marital status, beginning to work, and changing schools rank in the top three for the likelihood of sports engagement, with the last being a baby birth. As for recreational-WCP, changing jobs, changing marital status, and having a baby are the top three most likely to be involved, and changing schools is the last. Regarding dog walking, the top three life events are changing jobs, changing marital status, and stopping full-time education, and the last one is owning the first car.

According to the results, changes in marital status are important for participation in all three types of LTPA. This may imply the importance of social support from family members. Changes in marital status are always accompanied by changes in family structure, and previous research has documented that people are more likely to actively engage in LTPA under the influence of physically active significant others (Marquez & McAuley, 2006; Orsega-Smith et al., 2007). Additional significant life transitions are work-related, with people more likely to play sports when they start working and to participate in the other two LTPAs when they change jobs. The differences found may be related to age. Most people start working before the age of 23, in this age category people are more likely to play sports. While job changes typically occur after age 23 when people are more likely to engage in the other two LTPA types. Work-related events may contribute to LTPA engagement for several reasons, such as improved economic conditions, increased recreational time, more physically active workplaces and surroundings, etc.

#### 3.3.3 Neighborhood characteristics

With positive estimated values, greenspace for walking is significant for participation in LTPA types except for sports. That is, neighborhoods with plenty of walkable greenspaces are more supportive of people walking, cycling, and playing for recreation and walking their dogs than neighborhoods with insufficient walkable greenspaces. It's not surprising as greenspace is where these activities in general take place. Abundant greenspace means high accessibility, providing more opportunities for people to participate in these activities.

Cycling facilities are the only neighborhood characteristic significant to all the LTPA types. People living in neighborhoods with average or above-average cycling facilities have a higher probability of sporting and walking, cycling, and playing outside for pleasure but a lower probability of walking their dogs than in neighborhoods with below-average facilities. Besides, residents in neighborhoods with extensive cycling facilities are most likely to participate in recreational-WCP. In the Netherlands, cycling is not only a mode for daily travel but also a popular for sport and entertainment. If the neighborhood bicycle facilities are adequate, people may increase their use of bicycles. On the one hand, it naturally raises their opportunities of choosing cycling as a sport or recreational activity. On the other hand, it allows easy access to sports venues, thereby increasing the possibilities of sporting.

Supportive facilities for physical activities are significant to dog walking and recreational-WCP. Neighborhoods with lots of physical activity facilities are most likely to promote dog walking. While recreational-WCP are estimated to be most likely to occur among residents of neighborhoods with average or above-average physical activity facilities, followed by below-average neighborhoods, but are least likely to occur in neighborhoods with lots of physical activity facilities. One possible explanation is that numerous facilities for physical activities can enrich residents’ activity forms, and recreational-WCP may not be their first choice.

Regarding safety for physical activity, people in above-average neighborhoods are more likely to engage in recreational-WCP but less likely to walk their dogs than those in average or below-average scoring neighborhoods. It may be explained by people's instinctive response to risk aversion, i.e., safety concerns may reduce people's interest in going out for leisure. As for dog walking, it may be related to the demographics of different neighborhoods. As discussed earlier, lower educated people are more likely to walk their dogs, and such population group is more likely to live in average or below-average safe neighborhoods. Of course, it could also be due to relatively small sample size for dog walking (see Table 3).

#### 3.3.4 Time-related factors

The study-related physical activity time variable showed that students are more likely to play sports when they spend more than 2.5h/w on physical education and active commuting and more likely to walk their dogs when they spend less than 2.5h/w. One explanation is that students with longer durations of study-related physical activity are usually in primary or secondary schools. At that time, there is not much pressure to study, and students can devote more time to leisure activities like sports. Another explanation is that students with longer durations of study-related physical activity are inherently more enthusiastic about sports and are willing to invest more time in them.

Estimated results of work-related physical activity time highlight its role in sports participation. People are more likely to do sports when they spend less than 2.5h/w of physical activity during work and active commuting but less likely to do sports when they spend more than 5h/w. It may be occupation related. Kirk and Rhodes (2011) pointed out that blue-collar workers, who require more physical effort at work, tend to be less active in LTPAs. Also, it may be associated with remaining leisure time. People with lots of commuting time may have little leisure time.

#### 3.3.5 Translation parameters

The final row of Table 5 provides the estimated values of the translation parameters γ_k_ and the corresponding p-values. Serving as satiation parameters, γ_k_ (*k* = 2, 3, or 4) influences the time investment in each LTPA. According to Bhat (2005, 2008), a value of γ_k_ closer to zero for alternative k implies higher satiation (or lower time investment), while a high value implies lower satiation (or higher time investment). As shown, the γ_k_ results of this study are significantly different from 0, thereby reflecting different levels of satiation effects based on LTPA types. A high satiation effect (low duration) is observed in recreational-WCP, whereas a low satiation effect (high duration) is observed in sports. The satiation effect for dog walking is somewhere in between, closer to recreational-WCP.

## 4 Conclusions and discussion

This study applied an MDCEV model to jointly analyze the determinants of habitual participation in and time allocation to multiple LTPA types over the life course. This modelling system considered a comprehensive set of sociodemographic variables, life transitions, neighborhood characteristics, and time-related factors as potential determinants of habitual LTPA behavior. LTPA behavior is explored by activity type: sports, recreational-WCP, and dog walking, with durations measured in hours per week. Data was collected among a panel of 627 adult panelists in the Netherlands through an online retrospective survey. The model estimation findings expand our understanding of the determinants of each LTPA type from a life-trajectory perspective and can help to develop effective interventions to promote LTPA participation during life transitions.

This study provides several insights. Firstly, the influence of sociodemographic characteristics varies between LTPA types. All eight sociodemographic characteristics are relevant to sports participation. Sportive individuals are more likely to have one or more of the following characteristics: 7–23 years old, male, unpartnered, highly educated, owning a car, in good health, having family members with a chronic disease, living with physically active people. Five sociodemographic characteristics are associated with a greater likelihood of participation in recreational-WCP. These characteristics include being over 23 years old, having secondary or tertiary education, being chronically ill, having a family member with chronic diseases, and living with physically active people. Additionally, four characteristics affect dog walking. Individuals who are more than age 7, lowly educated, car owners, or chronically ill are more likely to walk their dogs.

Secondly, life transitions negatively affect all LTPA types, suggesting that people are less likely to keep on being engaged in LTPA when life transitions occur. Hence, interventions should be tailored to prevent declines in LTPA participation. According to the outcome comparisons for life transition variables, individuals are more likely to engage in recreational-WCP when life transitions occur. However, school transfers are an exception. When transfers occur, people are more likely to participate in sports. For different LTPA types, the impact of life transitions manifests differently. Changing marital status and work-related transitions appear important events for all LTPA types, as people are more likely to be involved when these events occur than when other transitions occur. Besides, changing schools is another major event that affects sports participation. Having children is also an especially significant event for recreational-WCP. While stopping full-time education is another particular event for dog walking.

Thirdly, neighborhood characteristics have a greater impact on recreational-WCP, and dog walking but less on sports. Specifically, physical activity-friendly neighborhoods are characterized by plenty of green spaces, lots of cycling facilities, average or above-average physical activity facilities, and high safety, and positively impact participation in recreational-WCP. Sports participation seems only affected by cycling facilities. People living in neighborhoods with average or above-average cycling facilities are more likely to play sports. Regarding dog walking, green spaces for walking and physical activity facilities appear to have positive effects, while cycling facilities and safety appear to have negative effects.

Finally, results of study-related physical activity time suggest that students are more likely to do sports when spending more than 2.5 h/w on physical education and active commuting. When spending less than 2.5h/w, they are more likely to walk their dogs. Additionally, work-related physical activity time only has a significant effect on sports participation. People are more likely to sport when spending less than 2.5h/w on physical activity during work and active commuting.

Note that this study has some limitations. One limitation is related to dog walking. Some results in this study of dog walking were a bit unexpected. These results may be correct or biased due to the small sample size. Further studies are needed to provide more evidence. Another limitation is about data collection. This study collected self-reported life-course data through a retrospective questionnaire. Such data may be somewhat limited due to recall errors or individual desirability. Although these possible biases have been minimized, risks are unavoidable. Despite these limitations, this study has displayed a more comprehensive and specific understanding of the determinants of different LTPA types from a life-course perspective. It can help architects, urban planners, sociologists, and public health practitioners to create a more supportive social and physical environment to promote healthy living.

## Ethics approval

This research was approved by the Ethical Review Board of the TU/e (Reference ERB2019BE2).

## CRediT author statement

**Xiaoyue Chen:** Conceptualization, Methodology, Investigation, Data Curation, Software, Writing - Original Draft. **Astrid Kemperman:** Conceptualization, Methodology, Investigation, Writing - Review & Editing, Supervision. **Harry Timmermans:** Conceptualization, Methodology, Investigation, Writing - Review & Editing, Supervision.

## Declaration of competing interest

None.

## Data Availability

The data that has been used is confidential.
